# Progressive myocardial injury in myotonic dystrophy type II and facioscapulohumeral muscular dystrophy 1: a cardiovascular magnetic resonance follow-up study

**DOI:** 10.1186/s12968-021-00812-6

**Published:** 2021-11-08

**Authors:** Edyta Blaszczyk, Carolin Lim, Peter Kellman, Luisa Schmacht, Jan Gröschel, Simone Spuler, Jeanette Schulz-Menger

**Affiliations:** 1 Department of Cardiology and Nephrology, Working Group Onn Cardiovascular Magnetic Resonance, Experimental and Clinical Research Center a Joint Cooperation Between the Charité – Universitätsmedizin Berlin, Department of Internal Medicine and Cardiology and the Max-Delbrueck Center for Molecular Medicine, and HELIOS Klinikum Berlin Buch, Lindenberger Weg 80, 13125 Berlin, Germany; 2 DZHK (German Centre for Cardiovascular Research), Partner Site Berlin, Berlin, Germany; 3 National Heart, Lung and Blood Institute, National Institute of Health, Bethesda, USA; 4 Muscle Research Unit, Experimental and Clinical Research Center a Jointoint Cooperationoperation Betweenetween the Charité Medical, Berlin, Germany

**Keywords:** Magnetic Resonance Imaging, Facioscapulohumeral muscular dystrophy type 1, Myotonic dystrophy type 2, Fat, Fibrosis, Remodeling

## Abstract

**Aim:**

Muscular dystrophy (MD) is a progressive disease with predominantly muscular symptoms. Myotonic dystrophy type II (MD2) and facioscapulohumeral muscular dystrophy type 1 (FSHD1) are gaining an increasing awareness, but data on cardiac involvement are conflicting. The aim of this study was to determine a progression of cardiac remodeling in both entities by applying cardiovascular magnetic resonance (CMR) and evaluate its potential relation to arrhythmias as well as to conduction abnormalities.

**Methods and results:**

83 MD2 and FSHD1 patients were followed. The participation was 87% in MD2 and 80% in FSHD1. 1.5 T CMR was performed to assess functional parameters as well as myocardial tissue characterization applying T1 and T2 mapping, fat/water-separated imaging and late gadolinium enhancement. Focal fibrosis was detected in 23% of MD2) and 33% of FSHD1 subjects and fat infiltration in 32% of MD2 and 28% of FSHD1 subjects, respectively. The incidence of all focal findings was higher at follow-up. T2 decreased, whereas native T1 remained stable. Global extracellular volume fraction (ECV) decreased similarly to the fibrosis volume while the total cell volume remained unchanged. All patients with focal fibrosis showed a significant increase in left ventricular (LV) and right ventricular (RV) volumes. An increase of arrhythmic events was observed. All patients with ventricular arrhythmias had focal myocardial changes and an increased volume of both ventricles (LV end-diastolic volume (EDV) p = 0.003, RVEDV p = 0.031). Patients with supraventricular tachycardias had a significantly higher left atrial volume (p = 0.047).

**Conclusion:**

We observed a remarkably fast and progressive decline of cardiac morphology and function as well as a progression of rhythm disturbances, even in asymptomatic patients with a potential association between an increase in arrhythmias and progression of myocardial tissue damage, such as focal fibrosis and fat infiltration, exists. These results suggest that MD2 and FSHD1 patients should be carefully followed-up to identify early development of remodeling and potential risks for the development of further cardiac events even in the absence of symptoms.

*Trial registration* ISRCTN, ID ISRCTN16491505. Registered 29 November 2017 – Retrospectively registered, http://www.isrctn.com/ISRCTN16491505

## Introduction

Muscular dystrophy (MD) is a group of genetic and progressive diseases with primary symptoms of skeletal muscle pain and weakness. In some MD, such as Duchenne muscular dystrophy (DMD) and Becker muscular dystrophy (BMD), cardiac involvement is well known. Myocardial fibrosis detected by cardiovascular magnetic resonance (CMR) enables the prediction of cardiac events in DMD/BMD patients independently when compared with reduced left ventricular (LV) ejection fraction (LVEF). Interestingly, in patients with preserved LVEF, there is added value of focal fibrosis [1–3]. Focal fibrosis may occur in up to 90% of these patients, leading to heart failure and sudden cardiac death (SCD) in some cases [4].

Myotonic dystrophy type II (MD2) and facioscapulohumeral muscular dystrophy type 1 (FSHD1) have gained an increasing awareness during the last years. There is a suspicion that MD2 as well as FSHD1 could be underdiagnosed due to frequently mild symptoms and slower progression in females. Late onset and a slower progression seem to lead to a rate of 20% misdiagnosed patients [5]. MD2 and FSHD1 are mainly recognized as muscular diseases with rare cardiac involvement.

MD2 is an autosomal dominant inherited multisystemic muscle disease. The mutation frequency constitutes 1:1830 [6]. Patients often notice the first symptoms quite late, at the age of 37 ± 15 years [7], suffering from muscle weakness, myotonia and muscle pain.

FSHD1 is an autosomal dominant disorder and the third most common inherited muscle disease with an incidence of 1: 8.000–1: 20.000 [8]. Diagnosis of FSHD1 is often suspected in patients with presence of progressive asymmetric weakness of the face and shoulder muscles. However, 10–25% of patients are wheelchair-dependent [9].

Arrhythmias in both patient groups are known but its relation to myocardial injury as well as evidence for progression of myocardial changes still remains unknown. Trevisan et al. reported arrhythmic events in 12% of FSHD-patients [10], whereas in MD2 different forms of arrhythmias were reported in 17% to 36% of patients [11].

In our pilot studies, we were able to identify myocardial injury, like fat infiltration and focal fibrosis, in over 26% of MD2 and FSHD1 patients with preserved LVEF [12, 13].

Due to individual predispositions, even mild initial dysfunction may lead to severe heart failure over months to years [14, 15]. However, systematic follow-up analysis in patients with MD2 and FSHD1 are lacking. Table 1 provides an overview of the most common muscular dystrophies and their associated cardiac abnormalities.

**Table 1:** Overview of muscular dystrophies and their associated cardiac abnormalities

Disease	Cardiologic manifestations				
	Clinical			CMR	
	Cardiac involvement (%)	Possible phenotype of the involvement	Conduction disturbances/ Arrhythmias	Extension of late gadolinium enhancement	Presence of fat infiltration
Duchenne muscular dystrophy	Up to 90	DCM	Sinus tachycardia, Ventricular tachycardias	+ + +	+
Becker muscular dystrophy	60–70	DCM	AV nodal and bundle branch blocks	+ + +	No data
Emery-Dreifuss muscular dystrophy	50–90	DCM, HCM, LVNC, biatrial dilation	Bradycardias (AV blocks), Tachycardias (SVTs)	Rare, if present associated with tachycardias	Rare
Limb girdle muscular dystrophy	25–90	DCM, HCM	SVTs, Ventricular tachycardia	+ +	+
Myofibrillar myopathy	40–60	DCM, HCM, LVNC	Complete AV Block	no data	No data
Facioscapulohumeral muscular dystrophy	5–25	DCM (rare)	RBBB, SVTs	+ / + +	+
Myotonic dystrophy type 1	60–80	DCM, HCM	AV Blocks, RBBB/LBBB QTc/QRS prolongation, PVC, Ventricular Tachycardia, Atrial fibrillation, Atrial flutter	+ +	No data
Myotonic dystrophy type 2	Up to 25	DCM, HCM	Atrial fibrillation	+ / + +	+

The aim of this study was to investigate a potential progression of the cardiac remodeling processes, including focal myocardial injury and function, in patients with MD2 and FSHD1 by applying serial CMR. Furthermore, we evaluated its relation to arrhythmias and conduction abnormalities.

## Methods

Follow-up was performed in 83 patients with genetically confirmed diagnosis of MD2 and FSHD1 who had previously participated in our studies [12, 13].

A detailed medical history was recorded including symptoms related to cardiovascular diseases, medication and cardiovascular risk factors. Known myocardial infarction or myocarditis were considered as exclusion criterion to avoid an overlap with myocardial injury due to a different cause. Blood pressure was taken before and after CMR. Assessment of heart rhythm abnormalities was based on a 12-lead electrocardiogram (ECG) and on 24 h ECG-monitoring. Patients were also considered at risk of SCD according to the Groh-criteria: no sinus rhythm, PR interval ≥ 240 ms, QRS duration ≥ 120 ms, second- or third-degree atrioventricular block [16].

Significant arrhythmias were defined as frequent premature ventricular contractions (PVC ≥ 1000 /24-h), episodes of non-sustained ventricular tachycardia (NSVT), runs of supraventricular tachycardia (SVT) and 2nd/3rd degree atrioventricular (AV) block.

The local university ethical board approved the study (EA1/042/17) and all subjects gave written, informed consent.

## Cardiovascular magnetic resonance

### CMR protocol

CMR was performed on a 1.5 T scanner (MAGNETOM AvantoFit®, Siemens Healthineers, Erlangen, Germany) using a 32-channel surface coil.

Cine imaging was performed applying a balanced steady state free precession (bSSFP) sequence to determine the global cardiac performance. We acquired the following long axis views for the LV: four chamber (4Ch), three chamber (3Ch) and two chamber (2Ch) views and for the right ventricle (RV) a single long axis view (echo time (TE) 1.2 ms; repetition time, 33 ms; voxel size 1.8 × 1.8 × 6.0 mm^3^) as well as a short axis (SAx) package (TE 1.2 ms; repetition time, 63 ms; voxel size 1.4 × 1.4 × 7.0 mm^3^) to cover the LV.

**Fig. 1:**
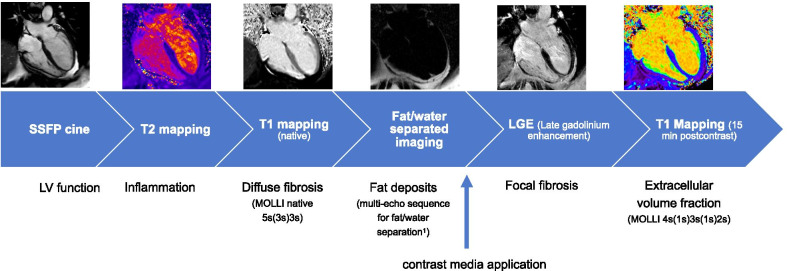
Scan protocol

For myocardial tissue differentiation parametric T1- and T2-mapping, fat/water-separated imaging and focal fibrosis imaging (late gadolinium enhancement, LGE) were acquired. An overview of the scan protocol is provided in Fig. 1.

A multi-echo sequence was used for fat/water-separation [17] in 4ch view and five SAx slices (gradient echo sequence (GRE), double inversion recovery dark blood preparation, four echoes with monopolar readout, TR 824 ms, TE 1.6–3.9–6.2–8.6 ms, slice thickness 6 mm).

We used the same contrast agent as in the previous studies (0.2 mmol/kg body weight of gadoteridol for MD2 and 0.15 mmol/kg body weight of gadobutrol for FSHD1). [12, 13].

LGE was performed in the same slice position as cine imaging in 4Ch, 3Ch, 2Ch views and SAx orientations (gradient echo sequence, breath-held segmented protocol with 10 ms echo spacing, TE of 5.2 ms, and slice thickness of 7 mm) 10–15 min after administration of contrast agent.

T2- and T1-mapping were performed in basal, mid and apical slices as described [12, 18]. Calculations were carried out for each segment and for each slice. Motion-corrected T2 mapping was based on a fast low angle shot (FLASH) gradient echo sequence in 4ch and SAx views as basal, mid-ventricular and apical slices. T2 maps were based on images with T2 preparation at times of 0/30/55 ms, and slice thickness of 6.0 mm, TR 251.49 ms and TE 1.32 ms.

Motion-corrected T1 mapping based on Modified Look-Locker Inversion Recovery (MOLLI) technique was performed before and 15 min after contrast media application using for T1 native: 5 s(3 s)3 s and for T1 post-contrast: 4 s(1 s)3 s(1 s)2 s pattern in 4Ch view and three SAx views with basal, mid-ventricular and apical slices (imaging parameters: TR = 281.64 ms (4ch) and 332.67 ms (SAX), TE = 1.12 ms, slice thickness 6.0 mm, GRAPPA acceleration factor 2).

### Data analysis

In the first paper (2016) we used cvi42 (version 4.1.2, Circle Cardiovascular Imaging, Calgary, Alberta, Canada). The analysis of the MD2 cohort at follow-up was performed in 2018/2019. At that timepoint, we switched to cvi42 (version 5.3.2, Circle Cardiovascular Imaging). Because of potential influences and possible inconsistencies between different software versions, a quality assurance test was performed (re-evaluation of the baseline results with the new version in a randomly chosen subgroup). There were no significant differences between the quantitative results. For the FSHD1 group we used the same version of Circle software (version 5.3.2, Circle Cardiovascular Imaging) for both baseline and follow up evaluations.

SAx cine images were used to determine LVEF and right ventricular (RV) ejection fraction (RVEF), volumes and LV mass by drawing endo- and epicardial contours (papillary muscles as part of the mass) at the end of the systolic and diastolic phases [19].

We quantified the left atrium (LA) area based on the biplanar approach using 2- and 4ch. For right atrium (RA) quantification 4Ch view cine images in LV systole were used. Furthermore, LA volume and ejection fraction were quantified based on the biplanar approach [20].

The quantitative analysis of mapping was performed as average value for slice as well as for each segment. The region-of-interest ROI was defined by the delineation of the endocardial and epicardial border of the myocardium. To ensure that blood or extra myocardial tissue were not included, endo- and epicardial offset of 5% was used. The segments were defined following the American Heart Association (AHA) segment model. The qualitative survey implied the exclusion of segments in case of artifacts (e.g., caused by susceptibility effects or unintended thoracic motion) or wrong motion correction.

The visual evaluation of the LGE images was performed by two independent, experienced readers (SCMR Level III) to assess presence, number and location of focal scars.

Quantification of LGE was performed with the established semi-automated signal threshold versus reference mean (STRM) method [21]. On all LGE images, endocardial and epicardial contours were manually traced and ROIs were defined in hyperenhanced and remote myocardium.

LGE was defined as myocardial signal intensity plus 3 standard deviations (SD) above remote, normal-appearing myocardium. The automated LGE detection could be manually corrected by the reader for a specific location to exclude obvious artifacts. After segmentation, myocardial and scar tissue (in %) were calculated [21, 22].

Fibrosis volume and the total cell volume were derived using extracellular volume fraction (ECV) and the following formulas [23]:

$$Fibrosis \, volume \, = \, ECV*LVmass/myocardial \, density*$$

$$Cell \, volume \, = \, \left( {\left( {1 \, - \, ECV} \right) \, *LV \, mass} \right)/myocardial \, density*$$

$$*myocardial \, density \, = \, 1.05 \, g/m.$$

Fat/water-separation imaging was analyzed using pre-defined criteria. A suspected region was considered positive if the intramyocardial fat was detectable (a) in the fat-separated image (hyperintense) and in the water-separated image (hypointense) or (b) detected in one of the separated images as well as in the cine imaging and LGE. Within the LV segmental analysis was performed following the AHA segment model [24].

Global longitudinal strain (GLS) using CMR feature tracking was assessed from the 4Ch, 3Ch, and 2Ch images. Endo- and epicardial contours were manually drawn in end-diastolic phase, defined as the phase with the largest LV volume. Trabeculae, papillary muscles, pericardium, and epicardial fat were consequently excluded from contouring [25].

### Statistical analysis

The statistical analysis was performed using SPSS® (version 25, Statistical Package for the Social Sciences, International Business Machines, Inc., Armonk, New York, USA). All results are shown as mean ± standard deviation. Normal distribution was analyzed graphically using the Kolmogorov–Smirnov test. For comparing FSHD1/MD2 patients before and after the follow-up period we used the Mann–Whitney-U test. A p value < 0.05 was considered to indicate a statistically significant difference. Intra- and inter-observer reproducibility was analyzed using intra-class correlation coefficient (ICC) and 95% confidence interval (CI). ICC was classified as poor (ICC < 0.4), good (ICC = 0.4–0.75) or excellent (ICC > 0.75). Images were analyzed twice by blinded readers.

## Results

### CMR analysis in MD2 and FSHD1 patients

Follow-up was available for 27 of 31 MD2 (87%) patients (follow up 3.9 ± 0.3 years) and 41 of 52 (80%) FSHD1 patients (follow up 2.0 ± 0.1 years). Six patients refused follow-up CMR. Twenty-two patients with MD2 and 40 subjects with FSHD1 underwent CMR with contrast. Baseline characteristics of patients at follow-up are shown in Tables 2 and 3. Some patients during the observation period received medications that could reduce the progression of cardiac remodeling and fibrotic processes: 10/22 MD2 and 7/41 FSHD1 patients received angiotensin converting enzyme (ACE) inhibitors or angiotensin receptor blockers (ARBs), only 3 patients in both groups received ß-blockers.

**Table 2:** Characteristics of patients with muscular dystrophy II (MD2) at follow-up

	Presence of Late Gadolinium Enhancement		
	All (n = 22)	No (n = 17)	Yes (n = 5)
Age *(years)*	58 ± 9	57 ± 9	61 ± 10
Male sex, n *(%)*	6 (27.3)	4	2
Heart rate *(beats per minute)*	69 ± 9	68 ± 9	72 ± 11
Systolic BP *(mmHg)*	130 ± 16	127 ± 16	139 ± 16
Diastolic BP *(mmHg)*	75 ± 10	73 ± 10	81 ± 9
Arterial hypertension, n *(%)*	7 (31.8)	4	3
Hyperlipidemia, n *(%)*	16 (72.7)	13	3
Diabetes mellitus, n *(%)*	3 (13.6)	3	–
Smoking, n *(%)*	2 (9.1)	1	1
Cardiac symptoms			
Asymptomatic, n (%)	16 (72.7)	11	2
Palpitations, n (%)	6 (27.3)	4	2
Chest pain, n (%)	1(4.5)	1	0
Fatigue, n (%)	5 (22.7)	4	1

Data are shown as mean values ± standard deviation (SD) according to the AHA-segment model. *BP* blood pressure

**Table 3:** Characteristics of patients with Facioscapulohumeral muscular dystrophy type 1 (FSHD1) at follow-up

	Presence of Late Gadolinium Enhancement		
	All (n = 40)	No (n = 26)	Yes (n = 14)
Age *(years)*	49 ± 14	47 ± 14	54 ± 11
Male sex, n *(%)*	29 (70.7)	9 (36)	3 (21.4)
Heart rate *(beats per minute)*	74 ± 13	76 ± 13	71 ± 14
Systolic BP *(mmHg)*	129 ± 15	127 ± 16	132 ± 15
Diastolic BP *(mmHg)*	79 ± 10	79 ± 9	79 ± 11
Arterial hypertension, n *(%)*	8 (20)	1	7
Hyperlipidemia, n *(%)*	7 (17.5)	3	4
Diabetes mellitus, n *(%)*	3 (7.5)	1	2
Smoking, n *(%)*	5 (12.5)	3	2
Cardiac symptoms			
Asymptomatic, n (%)	29 (70.7)	19	10
Palpitations, n (%)	8 (12.2)	6	2
Chest pain, n (%)	1(2.4)	1	0
Fatigue, n (%)	6 (14.6)	4	2

Data are shown as mean values ± standard deviation (SD) according to the AHA-segment model. *BP* blood pressure

## Myotonic dystrophy type II

**Fig. 2:**
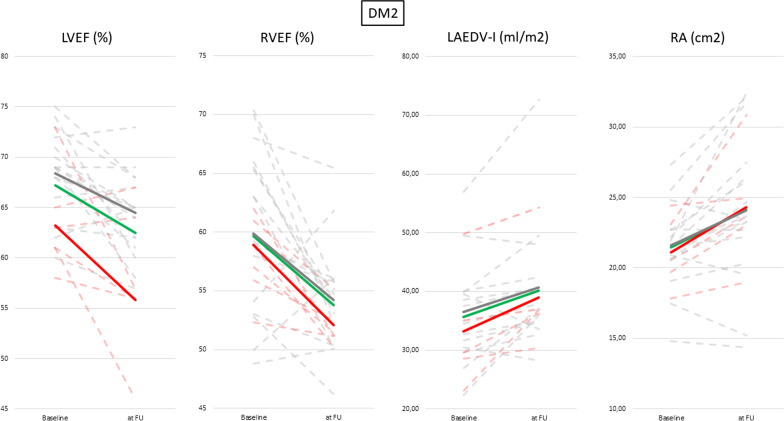
Changes of atria and ventricle parameters between baseline and follup in muscular dystrophy II (MD2) patients. Continuous lines are showing average values for: patients with focal fibrosis (red line), patients without focal fibrosis (grey line) and average value of all patients (green line) at baseline and follow-up. Dashed lines are presenting the values for each individual: with focal fibrosis (red line) and patients without focal fibrosis (grey line)

Individual and mean changes between both baseline and follow-up groups are displayed in Fig. 2.

### Remodeling

#### Left ventricular and right ventricular chamber anatomy

LVEF stayed within normal range, however was statistically lower at follow up (LVEF_baseline_ 68 ± 6% vs LVEF_FU_ 62 ± 6%, p = 0.001). After a mean observational period of four years, MD2 patients presented with significantly lower and mildly impaired RVEF compared to their baseline examinations (RVEF_baseline_ 59 ± 7 vs RVEF_FU_ 54 ± 4%, p = 0.001). Both ventricles showed no significant changes in volume during the course of time (LV end-diastolic volume (LVEDV) p = 0.605 and indexed LVEDV (LVEDVI) p = 0.275, RVEDV p = 0.444 and RVEDVI p = 0.456.) (Table 4).

**Table 4:** CMR parameters of patients with MD2 and FSHD1 at baseline and at follow-up

Parameter	MD2			FSHD1		
	Baseline (n = 31)	Follow-up n = 22	p-value	Baseline n = 52	Follow-up n = 41	p value
LVEF *(%)*	68 ± 6	62 ± 6	** < 0.01**	63 ± 5	60 ± 3	0.762
LVEDV *(ml)*	126 ± 22	124 ± 29	0.605	128 ± 21	139 ± 34	0.131
LVEDVI *(ml/cm)*	0.80 ± 0.11	0.73 ± 0.15	0.275	0.70 ± 0.10	0.79 ± 0.17	** < 0.01**
LV mass *(g)*	104 ± 27	92 ± 24	0.124	99 ± 25	102 ± 24	0.630
LV mass index *(g/cm)*	0.60 ± 0.14	0.54 ± 0.12	0.110	0.56 ± 0.13	0.57 ± 0.12	0.524
LV stroke volume (*ml)*	85 ± 13	78 ± 17	0.113	80 ± 15	84 ± 18	0.084
LV stroke volume index *(ml/m*^*2*^*)*	46 ± 6	42 ± 6	0.063	45 ± 8	48 ± 9	0.245
RVEF *(%)*	59 ± 7	54 ± 4	** < 0.01**	51 ± 6	49 ± 5	0.104
RVEDV *(ml)*	140 ± 29	146 ± 33	0.444	160 ± 31	169 ± 28	0.470
RVEDVI *(ml/m*^*2*^*)*	76 ± 15	79 ± 14	0.456	83 ± 16	88 ± 14	0.422
RV stroke volume *(ml)*	82 ± 18	80 ± 19	0.932	76 ± 18	83 ± 18	0.082
RV stroke volume index *(ml/m*^*2*^*)*	44 ± 89	43 ± 8	0.852	40 ± 8	43 ± 8	0.063
LAEF *(%)*	60 ± 8	57 ± 7	0.392	62 ± 8	60 ± 7	0.452
LAEDV *(ml)*	63 ± 18	71 ± 25	0.275	54 ± 14	61 ± 14	**0.021**
LAEDVI*(ml/m*^*2*^*)*	35 ± 8	38 ± 25	0.288	28 ± 7	32 ± 8	**0.027**
LA *(cm*^*2*^*)*	21 ± 3	24 ± 5	** < 0.01**	19 ± 3	22 ± 3	** < 0.01**
RA *(cm*^*2*^*)*	21 ± 3	24 ± 5	**0.040**	20 ± 3	23 ± 4	**0.029**
Native T1 basal *(ms)*	1029 ± 30	1015 ± 38	p = 0.066	1010 ± 26	991 ± 24	** < 0.01**
Native T1 mid *(ms)*	1012 ± 38	998 ± 30	p = 0.258	991 ± 39	989 ± 30	p = 0.102
Native T1 apical *(ms)*	1018 ± 50	999 ± 32	p = 0.163	983 ± 41	970 ± 40	p = 0.203
ECV basal *(%)*	26 ± 3	24 ± 3	** < 0.01**	26 ± 3	22 ± 2	** < 0.01**
ECV mid *(%)*	26 ± 2	24 ± 2	** < 0.01**	26 ± 3	23 ± 3	** < 0.01**
ECV apical *(%)*	29 ± 3	26 ± 2	** < 0.01**	27 ± 3	24 ± 2	** < 0.01**
T2 basal *(ms)*	51 ± 2	49 ± 2	** < 0.01**	50 ± 4	47 ± 2	** < 0.01**
T2 mid *(ms)*	52 ± 3	49 ± 3	** < 0.01**	51 ± 3	47 ± 2	** < 0.01**
T2 apical *(ms)*	55 ± 4	50 ± 2	** < 0.01**	53 ± 3	48 ± 2	** < 0.01**
Cell volume basal *(ml)*	72 ± 18	68 ± 17	p = 0.409	71 ± 19	74 ± 22	p = 0.308
Cell volume mid *(ml)*	70 ± 19	76 ± 17	p = 0.553	71 ± 20	73 ± 22	p = 0.361
Fibrosis volume basal *(ml)*	25 ± 9	20 ± 7	p = 0.055	24 ± 8	20 ± 7	**p = 0.023**
Fibrosis volume mid *(ml)*	26 ± 7	21 ± 6	** < 0.01**	23 ± 7	22 ± 7	**p = 0.032**
GLS *(%) LGE(-) patients*	-17.9 ± 1	-16.8 ± 1	** < 0.01**	-18.3 ± 1	-16.4 ± 1	**p < 0.01**

Data are shown as mean values ± standard deviation (SD) according to the AHA-segment model. Significant differences (p < 0.05) are highlighted in bold

*BMI* body mass index, BSA body surface area (Mosteller), *HR* heart rate, *BP* blood pressure, *LVEF* left ventricular ejection fraction, *LVEDV *left ventricular end-diastolic volume, *LVEDVI *eft ventricular end-diastolic volume index, *LVESV* left ventricular end-systolic volume, *LA * left atrium, *RA* right atrium, *GLS* global longitudinal strain, *ECV* extracellular volume fraction

At follow up patients with focal fibrosis showed significantly lower LVEF (LVEF_LGE+_ 59 ± 2% vs LVEF_LGE-_ 64 ± 4%, p = 0.005), however it still remained in normal ranges. LVEDV and LVEDVI did not change at follow up (LVEDV_LGE+_ 142 ± 38 vs LVEDV_LGE-_ 119 ± 25 ml p = 0.127, LVEDVI_LGE+_ 0.81 ± 0.19 ml/m^2^ vs LVEDVI_LGE-_ 0.70 ± 0.13, p = 207).

#### Left atria and right atria quantification

We observed significant increase of the LA and RA areas (LA _baseline_ 21 ± 3 vs LA _FU_ 24 ± 5 cm^2^, p = 0.014, RA _baseline_ 21 ± 3 vs RA _FU_ 24 ± 5 cm^2^, p = 0.040), however LA volume showed no significant changes (for LAEDV p = 0.275 and for LAEDVI p = 0.288) (Table 4).

We observed an excellent intra- and inter-observer reproducibility for ventricular and atrial assessment in both groups. ICC was 0.091 for intra-observer and 0.892 for inter-observer analysis.

### Myocardial tissue differentiation

#### Parametric mapping, cell and fibrosis volume

We performed T2 and T1 mapping at baseline and during follow-up. Native T1 values remained stable (MD2: basal p = 0.066, mid p = 0.258, apical p = 0.163), but ECV dropped significantly (MD2: basal p = 0.014, mid p < 0.01, apical p < 0.01). T2 decreased significantly as well (MD2: basal p < 0.01, mid p < 0.01, apical p < 0.01).

While the cell volume remained constant, patients at follow-up presented with lower fibrosis volume (MD2 cell volume basal p = 0.409, mid p = 0.553; fibrosis volume basal p = 0.055, mid p = 0.009) (Table 4).

#### Focal fibrosis and its relation to cardiac remodeling

**Fig. 3:**
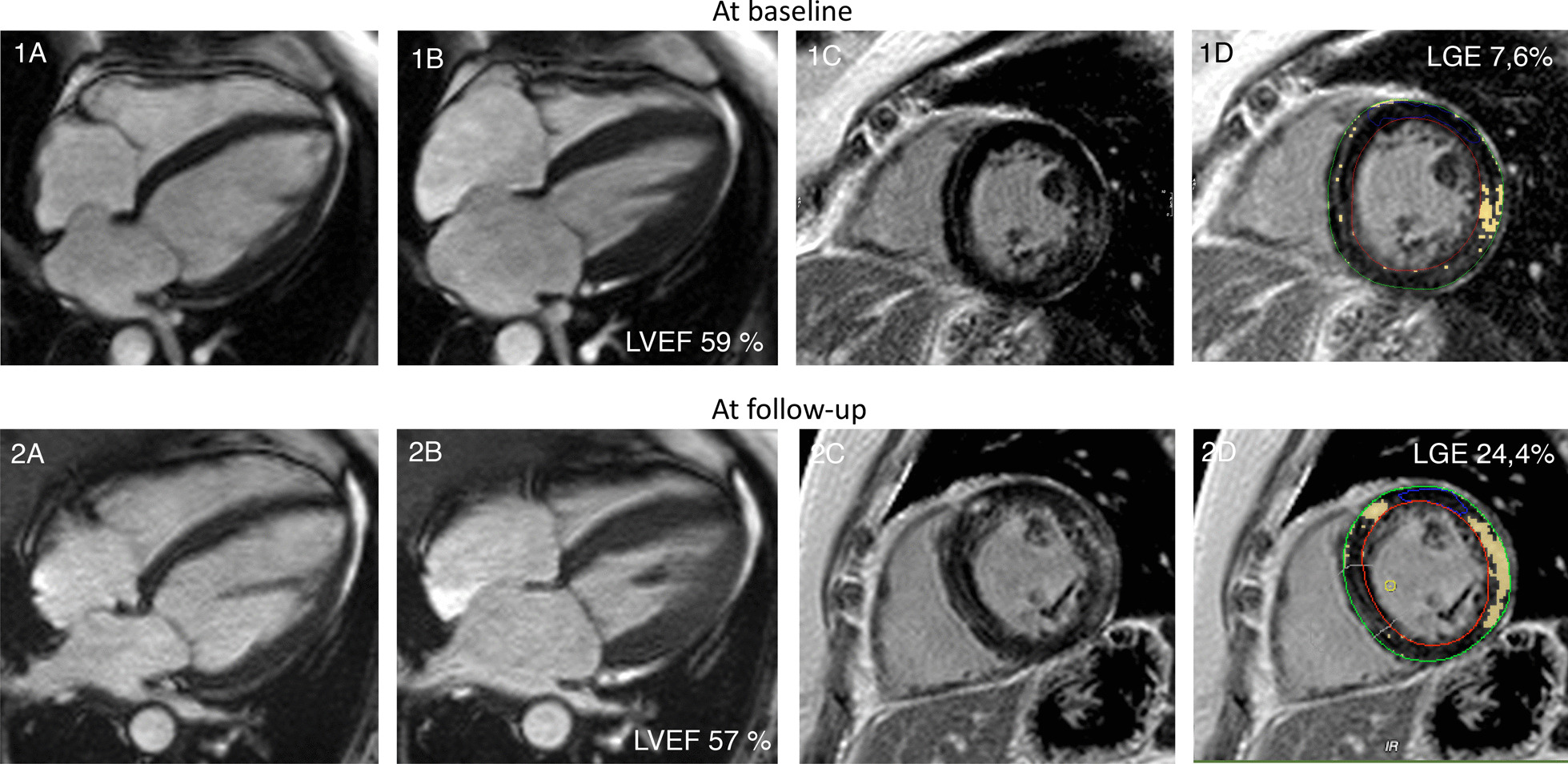
MD2 patient with progressive intramyocardial fibrosis and conduction abnormalities (atria-ventricular (AV) Block I, left anterior hemiblock) and still preserved ejection fraction. **1A**–**1D** at baseline. **2A**–**2D** at follow-up. Cine images in 4ch in diastole (**1**, **2 A**) and systole (**1**,**2 B**). Late gadolinium enhancement (LGE) in short axis views (**1**, **2** **C**, **D**)

In the MD2 group, new focal fibrosis could be identified in 1 of 22 patients, (5%, female) at follow up. It was located in the basal segments of the inferolateral and inferoseptal wall. At follow-up, an increase of focal fibrosis was observed. In total 6/22 (27%,3 women) MD2 patients had focal fibrosis (see Fig. 3 and Table 5).

**Table 5:** Clinical characteristics and imaging findings according to the distribution of late gadolinium enhancement (LGE) and fat infiltration in DM2 and FSHD1 patients at baseline and at follow-up

	All DM2 patients		DM2 LGE (+)		DM2 Fat (+)	
Type of Arrhythmias	Baseline (n = 32)	Follow-up n = 22	Baseline (n = 5)	Follow-up n = 6	Baseline n = 6	Follow-up n = 7
SVT (n)	2	9	–	5	–	5
Non-sustained VT n (n)	–	–	–	–	–	–
Frequent PVC (≥1000/24h) (n)	–	–	–	–	–	–
AV Block I (n)	4	5	2	2	2	2
AV Block II (n)	–	1	–	–	–	–
LBBB	1	1	1	1	1	1
RBBB	1	2	–	1	–	–
LAH	2	3	–	1	–	–
*LGE location and global volume*			5	6		
Inferolateral basal (n)*)*	5	6	5	6	1	3
Anterolateral basal (n)	–	2	–	2	–	–
Septal (n)	1	1	1	1	–	–
LGE area - mean (%)			8.4	17.6		
Fat apical					6	7
*Medical therapy*						
Beta blockers (n)	1	3	–	1	–	1
ACE, Sartans (n)	4	10	1	2	–	2

	All FSHD1 patients		FSHD1 LGE (+)		FSHD1 Fat (+)	
Type of Arrhythmias	Baseline (n = 52)	Follow-up (n = 40)	Baseline (n = 13)	Follow-up (n = 15)	Baseline (n = 7)	Follow-up (n = 12)
SVT (n)	1	8	1	3	–	5
Non-sustained VT n (n)	1	2	1	2	1	1
Frequent PVC (≥1000/24h) (n)	2	11	1	8	2	3
AV Block I (n)	1	3	1	1	–	1
AV Block II (n)	–	–	–	–	–	–
LBBB	–	–	–	–	–	–
RBBB	–	–	–	–	–	–
LAH	–	–	–	–	–	–
*LGE location and global volume*			13	15	2	3
Inferolateral basal (n)*)*	7	7	7	7	–	–
Anterolateral basal (n)	2	1	2	1	–	–
Septal (n)	3	3	3	3	–	–
Inferior (n)	1	3	1	3	–	–
LGE area - mean (%)			18.6	28.9		
Fat apical					7	12
*Medical therapy*						
Beta blockers (n)	1	4		1	1	3
ACE-I, ARBs (n)	2	7	1	3	1	2

SVT = supraventricular tachycardia, VT = ventricular tachycardia, PVC = premature ventricular contractions, LBBB = left bundle branch block, RBBB = right bundle branch block, LAH = left anterior hemiblock, LGE = late gadolinium enhancement. ACE-I = angiotensin-converting enzyme (ACE) inhibitors, ARBs = angiotensin II receptor blockers

#### Focal fat

**Fig. 4.**
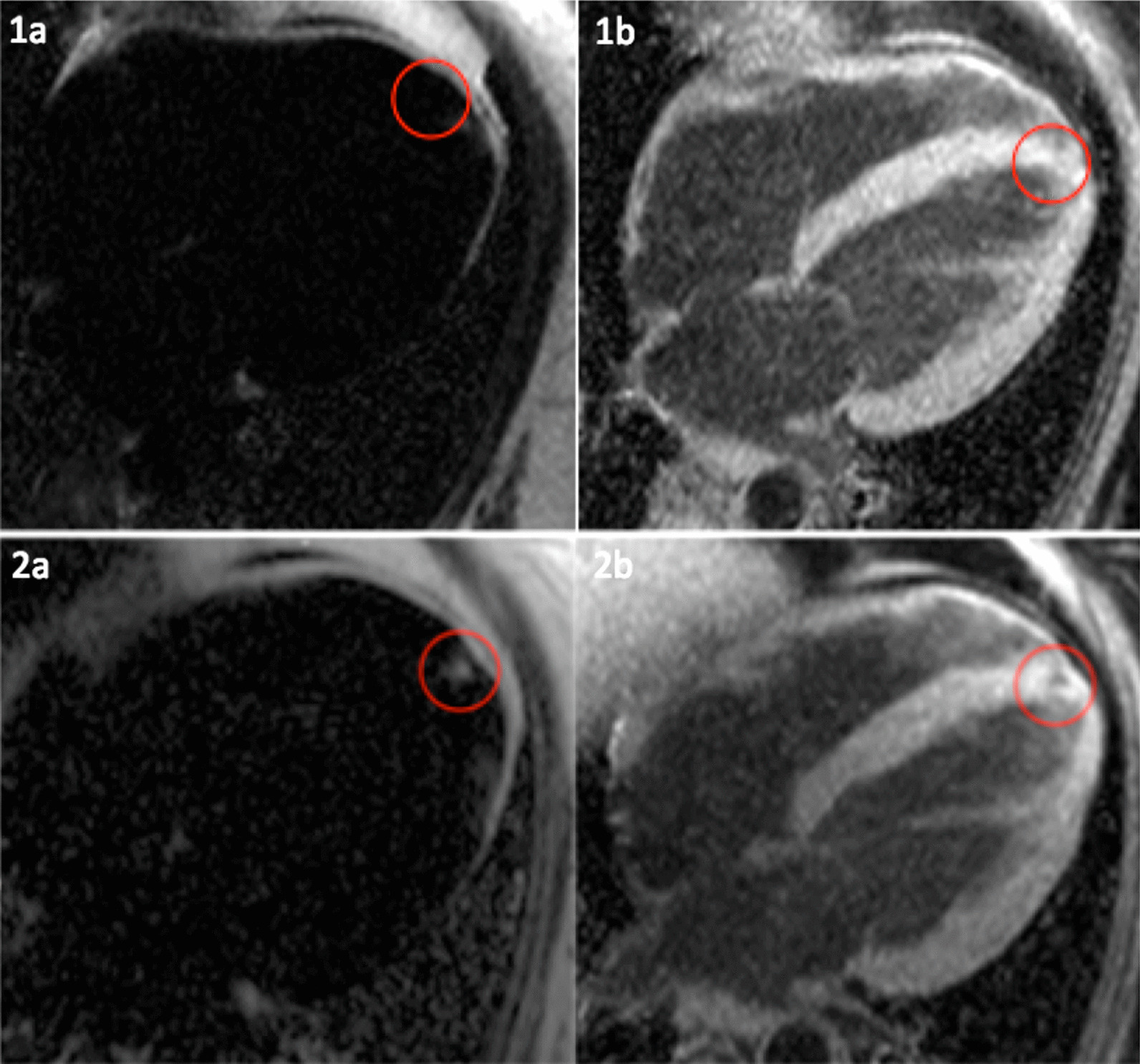
4-chamber view. Patient with MD2 and new apical fat infiltration. CMR 1: fat-separated image (**1a**) and water-separated image (**1b**) without evidence of fat infiltration. CMR 2: new apical fat infiltration, bright in the fat-separated image (**2a**) and hypointense (**2b**) in the water-separated image

New focal fat infiltration was observed in 2 of 22 patients (10%, both females), mostly located in the apical part of the interventricular septal wall (Fig. 4). Overall, fat infiltration was present in 7/22 (32%, all females) patients at follow-up.

#### Myocardial deformation- global longitudinal strain (GLS)

GLS was significantly lower in MD2 patients at follow in comparison to baseline (GLS_MD2 LGE (-) baseline_ -17.9 ± 1.0% vs. GLS_MD2 LGE (-) at follow up_ -16.8 ± 4.0%, p < 0.01). LGE ( +) patients were excluded to avoid the influence of known focal fibrosis.

#### Heart rhythm abnormalities and its relation to myocardial tissue changes

12-lead ECG was available in all patients, Holter-ECG in 24/27 patients.

New arrhythmic events or conduction abnormalities were recorded in 10/27 patients (37%). New episodes of SVT occurred in 7 patients while a new AV block type 2 was detected in only 1 patient. Two patients displayed new conduction abnormalities, specifically right bundle branch block and left anterior hemiblock. Positive Groh-criteria (AV block type 1) could be identified in only one patient. Arrhythmias or conduction disturbances were observed in all seven patients with fatty infiltrations and 4 of 6 patients with focal fibrosis. See Table 5.

## Facioscapulohumeral Muscular Dystrophy 1 (FSHD1)

**Fig. 5:**
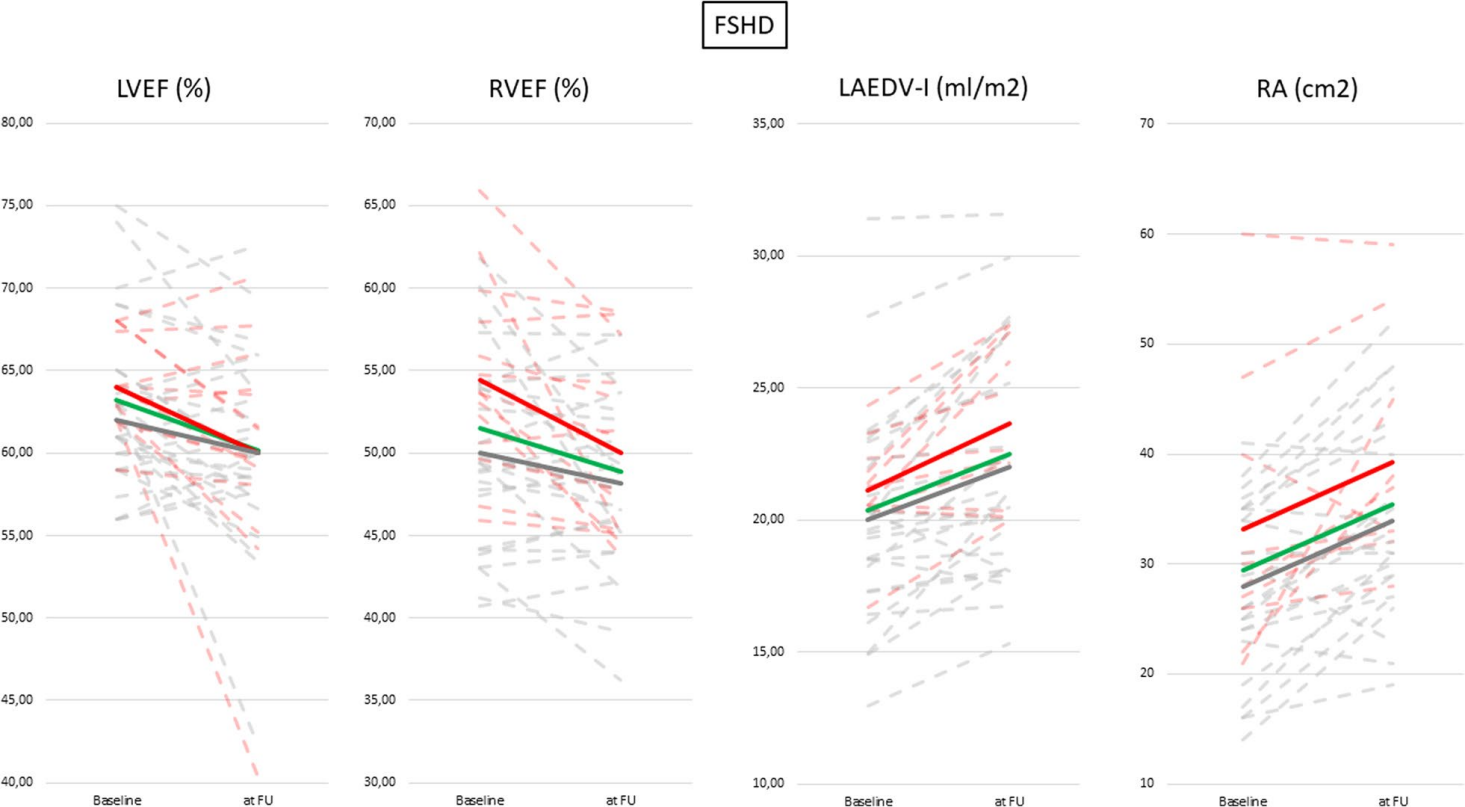
Changes of atria and ventricle parameters between baseline and at follow up in FSHD1 patients. Continuous lines are showing average values for: patients with focal fibrosis (red line), patients without focal fibrosis (grey line) and average value of all patients (green line) at baseline and follow up. Dashed lines are presenting the values for each individual: with focal fibrosis (red line) and patients without focal fibrosis (grey line)

Individual and mean changes between baseline and follow-up in both groups are displayed in Fig. 5.

### Remodeling

#### Left ventricular and right ventricular chamber size and function

During the follow up period, LVEF remained within the normal range (LVEF_baseline_ 63 ± 5% vs LVEF_FU_ 60 ± 3%, p = 0.762). There was no significant progression of RV dysfunction, however RVEF in FSHD1 patients was mildly impaired (RVEF_baseline_ 51 ± 6 vs RVEF_FU_ 49 ± 5%, p = 0.001). Volume in both ventricles stayed within normal range during the course of time, however LVEDVI increased significantly at follow-up (LVEDV p = 0.131 and LVEDVI p = 0.013, RVEDV p = 0.470 and RVEDV-I p = 0.422; Table 4).

#### Quantification of the left atria and right atria size

We observed a significant progression of both LA and RA areas (LA _baseline_ 19 ± 3 vs LA _FU_ 22 ± 3 cm^2^, p < 0.001, RA _baseline_ 20 ± 3 vs RA _FU_ 23 ± 4 cm^2^, p = 0.029) as well as LA volume (for LAEDV p = 0.271 and for LAEDVI p = 0.227).

### Myocardial tissue differentiation

#### Parametric mapping, cell and fibrosis volume

We performed T2 and T1 mapping at baseline and during follow-up. Native T1 values dropped in basal slices but remained stable in mid and apical slices (FSHD1: basal p = 0.001, mid p = 0.102, apical p = 0.203). ECV dropped significantly within three whole slices (basal p < 0.001, mid p < 0.001, apical p < 0.001). T2 mapping values decreased significantly similarly as in MD2 patients (basal p < 0.001, mid p < 0.001, apical p < 0.001). Interestingly, we observed the same phenomenon to that in MD2 patients regarding the cell and fibrosis volume. While the cell volume remained constant, patients at follow-up presented a statistically lower volume of fibrosis (cell volume p = 0.306, mid p = 0.361, fibrosis volume basal p = 0.023, mid p = 0.032). We included detailed information in the table with the CMR parameters (Table 4).

#### Focal fibrosis and its relation to cardiac remodeling

**Fig. 6.**
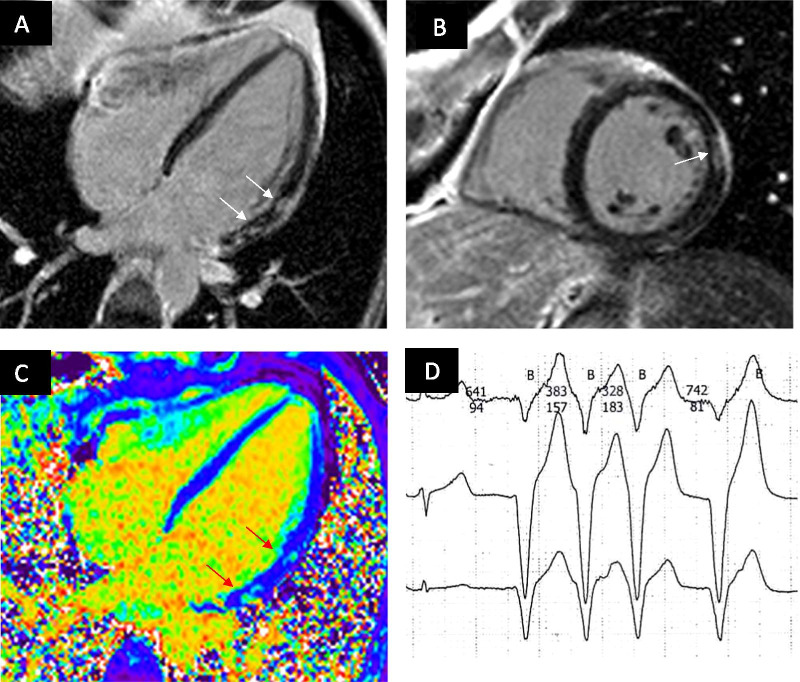
34-year-old asymptomatic man with FSHD1 presenting short episode of non-sustained ventricular tachycardia (VT) in Holter-ECG (**4D**) and evidence on LGE-CMR (LGE in 4Ch **4A** and short axis **4B**) involving LV lateral wall. Extracellular volume fraction (ECV) map in 4ch **4C**

In the FSHD1 group, new focal fibrosis was detected in 2 of 40 patients (6%, male). The fibrosis was located inferolateral and inferoseptal. Overall, in 15 of 40 (37%, 3 females) patients focal fibrosis could be identified. The pattern of the fibrosis was non-ischemic with an intramural and subepicardial distribution (Fig. 6). During the follow-up we also observed a quantitative increase in focal fibrosis (Table 5).

At follow up, patients with focal fibrosis showed no significant changes in LVEF (LVEF_LGE+_ 60 ± 7% vs LVEF_LGE-_ 60 ± 6%, p = 0.356), as well as in LVEDV and LVEDVI (LVEDV_LGE+_ 152 ± 46 vs LVEDV_LGE-_ 132 ± 23 ml p = 0.242, LVEDVI_LGE+_ 0.86 ± 0.23 ml/m^2^ vs LVEDVI_LGE-_ 0.74 ± 0.10, p = 131).

#### Focal fat

**Fig. 7.**
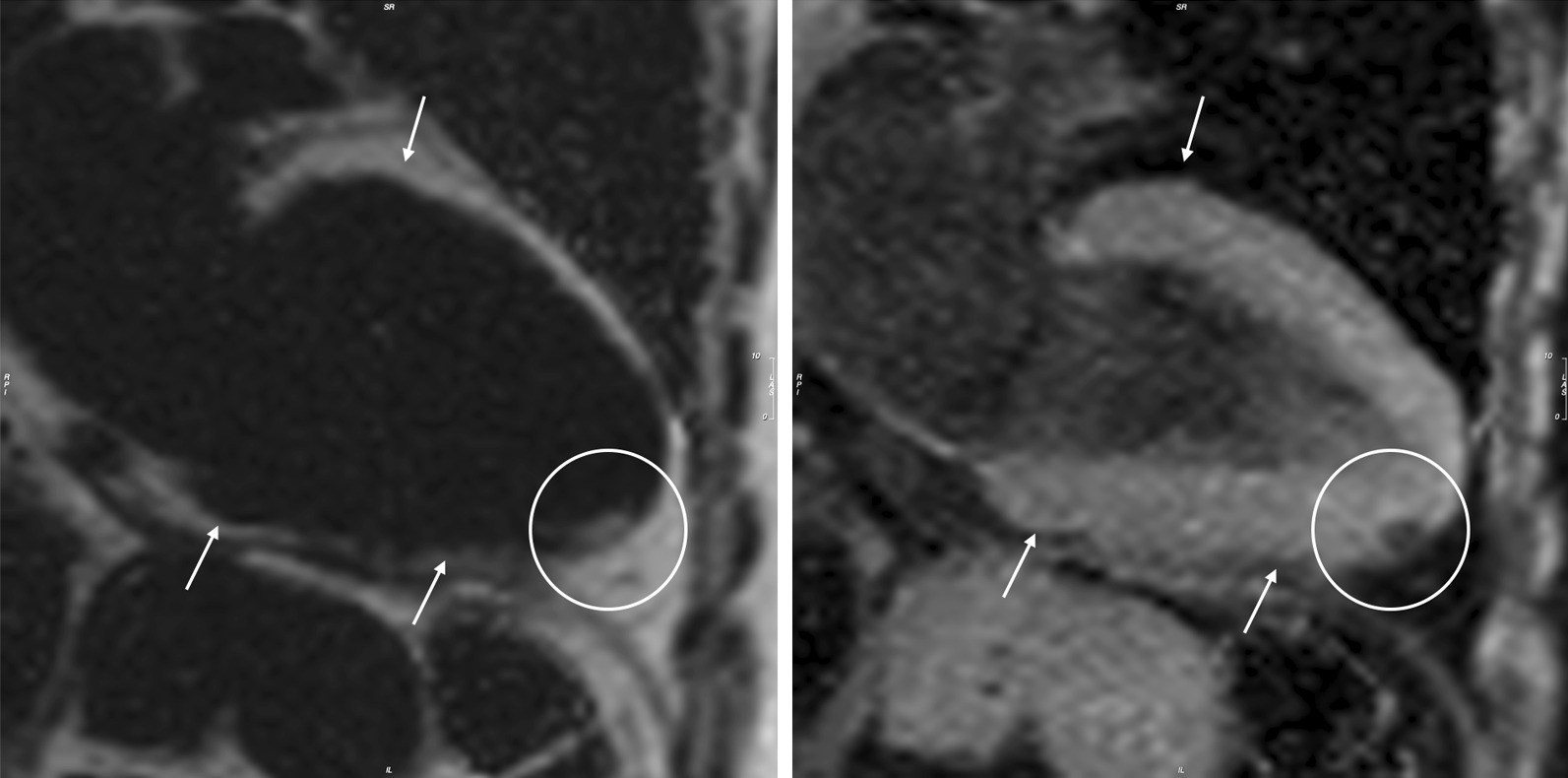
2-chamber view. Patient with FSHD1 and new intramyocardial apical fat infiltration- white circles. Bright in the fat-separated image (left) and hypointense (right) in the water-separated image. Epicardial fat – white arrows

New focal fat infiltration was observed in 5 of 40 patients (13%, 1 female). Majority was located in the apical part of the interventricular septal wall. In one patient the infiltration was found in the inferior wall (Fig. 7). Intramyocardial fat could be detected in 12 of 40 patients (30%, 3 female).

#### Myocardial deformation- global longitudinal strain (GLS)

To avoid the influence of known focal fibrosis, after exclusion of LGE ( +) patients, GLS was significantly lower in FSHD1 patients at follow in comparison to baseline (GLS_FSHD1 LGE (-) baseline_ -18.3 ± 1% vs. GLS_FSHD1 LGE (-) at follow up_ -16.4 ± 1%, p < 0.01).

#### Heart rhythm abnormalities and its relation to myocardial tissue changes

12-lead ECG was available in all patients, Holter-ECG in 34 of 40 patients (85%) with the diagnosis of FSHD1. The lack of data was due to patient related reasons.

New arrhythmic events were recorded in 10 of 34 patients (29%). NSVT was detected in one patient, runs of SVT in eight patients. Groh-criteria could not be identified. Holter was not available in 5/34 patients with focal fibrosis and/or fat infiltration. Ventricular arrhythmias (PVC > 1000) were observed in 8 patients with focal fibrosis and 3 patients with fat infiltration. SVTs were present in 5 patients with fatty infiltrations and 3 patients with focal fibrosis (Table 5).

## Discussion

In this study, we demonstrate that cardiac remodeling is progressive in both MD2 and FSHD1. Even in the absence of significant cardiac symptoms we observed a progression of structural and functional changes regarding all cardiac chambers.

The incidence of myocardial tissue changes such as focal fibrosis and fat infiltration was also higher at follow-up. There seems to be a relationship between structural abnormalities and abnormal heart rhythms and conduction abnormalities/disturbances. To the best of our knowledge, this is the first follow-up study applying CMR in patients with MD2 and FSHD1.

In inherited neuromuscular disorders such as DMD and BMD the development of a cardiomyopathy and/or heart failure is the second most important cause of death after respiratory failure. LV focal fibrosis was described in approximately 70% of these patients [26, 27]. The progression of myocardial fibrosis in other forms of MD are already well known [27, 28]. Furthermore, follow-up studies performed in DMD carriers showed progressive myocardial changes such as focal fibrosis and impaired LVEF [29]. In our study, we found that almost 30% of patients with MD2 and FSHD1 had focal fibrosis despite a preserved LVEF. However, in FSHD1 patients RVEF was mildly impaired starting already at baseline. During follow-up the remodeling of ventricle and atria was progressive. LV and RV functions worsened in both patient groups. Furthermore, we observed an increase of atrial size which was more evident in patients with known supraventricular arrhythmias. In almost 26% of patients with MD2, diabetes mellitus was already present at baseline. With this being a traditional cardiovascular risk factor associated with vascular events, it could contribute to cardiac remodeling, nevertheless all patients with focal fibrosis showed a non-ischemic LGE pattern.

Myocardial fat infiltration is a less studied matter in comparison to focal fibrosis as detection of fat by applying CMR is challenging. Thanks to recent technical developments identification of even small changes is feasible [17]. A correlation between fatty infiltration and arrhythmia frequency is already known in different diseases. Lu et al. reported the presence of myocardial fat in dilated cardiomyopathy (DCM) patients and its significant relationship to LV global function as well as a possible influence on the prognosis of DCM [30]. However, larger data sets are still missing and fatty remodeling seems to be underestimated and understudied. Fat within apical septum was reported also in healthy, however in our study focal fat infiltration was present in a much higher percentage of the studied cohort (30% of MD2 and FSHD1 patients). Especially in our MD2 patient group, all arrhythmic events were associated with the presence of fatty infiltrations, possibly underlying the impact on conduction abnormalities as discussed below.

In most patients with MD the LV is affected, presenting with a dilatation or reduced LVEF. However, over a span of the last few years there has been increasing awareness of the potential impact of RV impairment. RV and atrial remodeling may also dominate with clinical manifestations in neuromuscular diseases. These findings are often combined with rhythm- and conduction abnormalities [31]. Cardiac conduction abnormalities and atrial tachyarrhythmias are commonly observed in inherited MD and may also evolve from myocardial remodeling [32]. In patients with myotonic dystrophy type 1 (MD1), rhythm and conduction abnormalities are the dominant features of cardiac involvement, while heart failure seems to not be the most frequent finding in this entity. Asymptomatic MD1 patients with Groh-criteria were at higher risk of SCD when compared to those with normal ECGs. In Emery Dreifuss MD cardiac involvement is predominantly identified by conduction defects and atrial fibrillation/flutter. These patients often show atrial dilation in different stages [32, 33].

Patients with MD2 and FSHD1 are known to suffer from supraventricular arrhythmias as well as conduction disturbances like AV blocks [11]. In our group we observed the progression of atrial enlargement and a decrease of atrial function. This may explain the progression of SVT. Recently published studies have shown that multiple atrial premature contractions and SVTs predict stroke recurrence in patients with cryptogenic stroke without atrial fibrillation and may be a reproducible marker of atrial myopathy [33, 34]. Interestingly, Winterholler et al. showed an increased risk for ischemic strokes in DMD patients. It is suspected that cardioembolic stroke is an under-recognized complication in patients with MD [34–36].

Parametric mapping is a method that brings unique quantitative diagnostic information concerning the myocardium.

In our cohorts we observed an increase of the number of focal myocardial changes as well as a worsening of GLS. T2 decreased significantly. T1 values stayed stable in most segments while ECV dropped significantly in both groups. The correlation of the EVC to the LV-morphology showed, that meanwhile the cell volume stayed constant, the volume of fibrosis was lower at follow-up. The cause for the decrease remains speculative (possible cardioprotective medication, progressive fat infiltration?). We have further discussed progressive fat infiltration as a possible explanation, as one could expect especially in this disease, further remodeling in this direction. However, we refused this possibility because T2 decreased. The explanation remains speculative, but an influence of anti-remodeling medications and myocardial deoxygenation could explain the observed mapping variations. We assume, that the decrease of T2 can be explained by T2* effects and may reflect a deterioration of myocardial oxygenation that may play a role in the further development of fibrosis. It was previously shown, that lower T2* mapping values are related to alterations in the myocardial microcirculation. Manka et al. showed that BOLD CMR (blood oxygen level dependent) at rest revealed significantly lower T2* values for segments supplied by > 50% stenosed vessels [37]. Friedrich et al. presented the decrease of signal intensity during adenosine perfusion imaging within segments related to coronary artery stenoses > 75% [38]. Significant changes were also found in hypertensive patients compared to healthy controls [39].

We assume that the decrease of ECV at follow-up is probably related to a change of therapy between the two time points. The cardioprotective medication was optimized including ACE inhibitors. This may play a role in regards to the myocardial tissue changes within the whole group. Interestingly, Raman et al. could further show, that ACEI and mineralocorticoid receptor antagonists have an influence on fibrosis in MD [40]. There is no systematic CMR analysis investigating the effects of beta receptor blockade. This thesis is supported by our calculations, which show a reduced fibrosis volume with an unchanged total cell volume. Further follow-up trials will help to increase the understanding of these phenomenon’s and to define its impact on the patient prognosis.

Quantification of myocardial deformation is of growing interest in CMR. Although relatively new, CMR feature tracking has been performed in various myocardial diseases like cardiomyopathies, aortic valve diseases or myocardial infarction. It allows quantification of global and regional myocardial deformation offering additional information beyond ejection fraction and has the potential to detect subclinical myocardial dysfunction in patients with non-ischemic heart disease even in preserved ejection fraction and without wall motion abnormalities [41, 42].

In our cohort, GLS was significantly lower at follow up although global LV function was preserved. That could also be shown in patients without focal myocardial injury. It seems that a volumetric approach using LVEF may be less reliable during the first years of follow-up regarding the early phase of subclinical LV remodeling.

In our study we observed a remarkably fast progressive decline of the cardiac morphology and function as well as a progression of rhythm disturbances including arrhythmias, even in asymptomatic patients (see Fig. 5). Both ventricles as well as atria were affected. The changes occurred within 2–5 years. This underlines the need for routine ECG or echocardiographic testing even in asymptomatic patients. Currently, routine ECG and/or echocardiographic exams are only indicated in symptomatic FSHD1 patients.

Further multi-center follow-up studies are needed to understand the relation between cardiac remodeling in MD and the outcome. However, regular use of CMR for follow-up in these patients may provide a valuable risk stratification tool in the future.

## Limitations

Our sample size is relatively small, but both cohorts are recognized as rare diseases and this is the first follow-up study in this cohort. It was not possible to perform ECG-monitoring in all patients due to logistical reasons. This is an observational prospective cohort study, therefore there was no randomization into different treatment groups. Nevertheless, there is a potential impact of many other factors like progressive fat infiltration as well as the received therapy during the follow-up period, which should be taken into consideration. Currently, there are not enough data to report outcome analysis.

## Conclusions

Patients with MDs gain an increasing awareness in cardiology. We observed a remarkably fast progressive decline of cardiac morphology and function as well as a progression of rhythm disturbances including arrhythmias, even in asymptomatic patients. These changes occurred within a short period of time. It seems that a potential association between an increase of arrhythmias and progression of myocardial tissue damage such as focal fibrosis and fat infiltration exists. Our data suggest that these patients should be carefully followed to identify early development of remodeling and potential risks for the development of furthers cardiac events even in the absence of symptoms.

Longitudinal multi-center trials with a larger sample size will help to define the impact of our findings as well as further demonstrate a correlation between myocardial injury and arrhythmias in regards to long-term prognosis and therapeutic decision-making.

## Data Availability

The datasets used and/or analyzed during the current study are available from the corresponding author on reasonable request.

## Abbreviations

2Ch: Two chamber
3Ch: Three chamber
4Ch: Four chamber
ACE: Angiotensin converting enzyme
AHA: American Heart Association
ARB: Angiotensin receptor blocker
AV: Atrioventricular
BMD: Becker muscle dystrophy
bSSFP: Balanced steady state free precession
CMR: Cardiovascular magnetic resonance
DMD: Duchenne muscle dystrophy
ECG: Electrocardiogram
ECV: Extracellular volume fraction
FSHD1: Facioscapulohumeral muscular dystrophy type 1
GLS: Globla longitudinal strain
GRE: Gradient echo
LA: Left atrium/left atrial
LAEDV: Left atrial end-diastolic volume
LAEDVI: Left atrial end-diastolic volume index
LGE: Late gadolinium enhancement
LV: Left ventricle/left ventricular
LVEDV: Left ventricular end-diastolic volume
LVEDVI: Left ventricular end-diastolic volume index
LVEF: Left ventricular ejection fraction
MD: Muscular dystrophy
MD1: Myotonic dystrophy type 1
MD2: Myotonic dystrophy type 2
MOLLI: Modified Look-Locker inversion recovery
NSVT: Non-sustained ventricular tachycardia
PVC: Premature ventricular constrictions.
RA: Right atrium/right atrial
ROI: Region of interest
RV: Right ventricle/right ventricular
RVEDV: Right ventricular end-diastolic volume
RVEDVI: Right ventricular end-diastolic volume index
RVEF: Right ventricular ejection fraction
SAx: Short axis
SCD: Sudden cardiac death
STRM: Signal threshold versus reference mean
SVT: Supraventricular tachycardia
